# State Variability in Assisted Living Regulations, Access, and Outcomes for Persons With Dementia

**DOI:** 10.1093/geroni/igaa057.2522

**Published:** 2020-12-16

**Authors:** Kali Thomas

## Abstract

Approximately one million individuals, an estimated40% with a diagnosis of Alzheimer’s disease-related dementias (ADRD), reside in assisted living (AL); yet, little is known about their experience or the quality of care provided in AL. Unlike other forms of long-term care (LTC), the licensing, operating, and enforcement requirements for AL falls to the states, which vary dramatically in their regulatory approaches. The overall objective of this symposium is to examine states’ AL regulatory environments and understand if and how access to AL and the health outcomes of AL residents with ADRD are impacted by states’ regulatory decisions. Presenters will highlight the state variability in the regulation, access, and outcomes of AL residents with ADRD. The first presentation will describe the within and between state differences in regulatory approaches as it relates to dementia care. The second presentation will describe the variation in Medicaid financing of services in AL and its potential impact on access to AL within those states. The third will present geographic disparities in access to specialized dementia care in AL. The fourth presentation will characterize differences in emergency department utilization among AL residents with ADRD across states. Finally, the fifth presenter will report on the effect of establishing or increasing state staffing requirements on outcomes of AL residents with ADRD. Results will ultimately inform policy-makers, organizational leaders, and clinicians as they seek the most effective ways to ensure equal access to AL and optimal outcomes for residents with ADRD. Assisted Living Interest Group Sponsored Symposium.

